# *Gyre* and *gimble*: a maximum-likelihood replacement for Patterson correlation refinement

**DOI:** 10.1107/S2059798318001353

**Published:** 2018-04-03

**Authors:** Airlie J. McCoy, Robert D. Oeffner, Claudia Millán, Massimo Sammito, Isabel Usón, Randy J. Read

**Affiliations:** a Department of Haematology, Cambridge Institute for Medical Research, University of Cambridge, Hills Road, Cambridge CB2 0XY, England; b Crystallographic Methods, Institute of Molecular Biology of Barcelona (IBMB–CSIC), Barcelona Science Park, Helix Building, Baldiri Reixac 15, 08028 Barcelona, Spain; c ICREA, Institució Catalana de Recerca i Estudis Avançats, Passeig Lluís Companys 23, 08003 Barcelona, Spain

**Keywords:** Patterson correlation refinement, maximum likelihood, crystallographic phasing, fragment-based molecular replacement, antibodies

## Abstract

Descriptions are given of the maximum-likelihood *gyre* method implemented in *Phaser* for optimizing the orientation and relative position of rigid-body fragments of a model after the orientation of the model has been identified, but before the model has been positioned in the unit cell, and also the related *gimble* method for the refinement of rigid-body fragments of the model after positioning. *Gyre* refinement helps to lower the root-mean-square atomic displacements between model and target molecular-replacement solutions for the test case of antibody Fab(26-10) and improves structure solution with *ARCIMBOLDO_SHREDDER*.

## Abstract 2

Maximum-likelihood rigid-body refinement can be carried out to improve oriented models before the translation-function step of molecular replacement.

## 1. Introduction   

Brünger’s Patterson correlation (PC) refinement (Brünger, 1990 ▸) ascertained the value of breaking a molecular-replacement search model into smaller components and performing a refinement step between the traditional molecular-replacement rotation and translation functions at the point where only the orientation, but not the position, of the model is known. The principle of PC refinement is to take a list of possible orientations of a model, determined from a rotation function, divide the model into appropriate components, and then refine the orientation angles and relative translation coordinates of the components against the Patterson correlation target function (*i.e.* the correlation coefficient on structure-factor intensities). Starting separately from each of the orientations in the list, PC refinement itself may increase the signal of the rotation search sufficiently to make the correct orientation stand out from the noise, or with the rigid bodies correctly oriented and positioned relative to one another, the signal in the translation search may be much improved. PC refinement was first implemented in *X-PLOR* (Brünger, 1992 ▸) and subsequently in *CNS* (Brünger *et al.*, 1998 ▸), and has been highly cited in the crystallographic literature (Harzing & van der Wal, 2008 ▸). A brute-force search using the PC target was implemented in *BRUTE *(Fujinaga & Read, 1987 ▸).

The Patterson correlation is given by
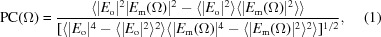
where the symbols 〈 〉 denote an averaging over the set of observed reflections expanded to *P*1, *E*_o_ denotes the normalized observed structure factors and *E*_m_ denotes the normalized calculated structure factors for the search model in orientation Ω and placed in the unit cell of the crystal with space group *P*1. Refinement of perturbations of the individual model orientations (Ω_*i*_) and relative translations (*t*_*i*_) from the overall orientation and original relative placement is performed by optimizing against
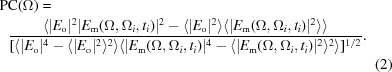



Although any parameterization of the model for PC refinement is possible, in practice PC refinement has been predominantly used with the model parameterized as rigid-body domains, where flexibility is expected between the model and target with respect to these domains. PC refinement has found particular favour with crystallographers who are tasked with solving crystal structures containing antibodies, where the hinge motion of the antibody makes molecular replacement challenging (Brünger, 1993 ▸). The implementations of PC refinement also allow the possibility of increasing the effective data-to-parameter ratio through the addition of a coordinate restraint term to the minimization target, in the form of an empirical energy function for geometric and nonbonded interactions (Brünger, 1990 ▸). Although used infrequently, this even allows the possibility of using PC refinement parameterized with the positions of individual atomic coordinates (Brünger, 1990 ▸).

A similar rotational refinement strategy was developed concurrently and independently by Yeates & Rini (1990 ▸). Two residual error functions were proposed as the target for refinement when only the orientation was known. Both of these include a sum over the intensities of the symmetry-related model structure factors, in contrast to PC refinement, which is performed with structure factors calculated from the model in a *P*1 cell identical in geometry to that of the crystal. Brünger showed that the inclusion of rotational symmetry in PC refinement simply increased the target function by a scale factor (Brünger, 1993 ▸). The second of the residual error functions proposed by Yeates and Rini differed from the first by down-weighting the unknown intermolecular vectors in Patterson space, the effect of which was similar to using normalized structure factors (*E* values) for PC refinement (Brünger, 1993 ▸). Subsequently, other target functions for PC refinement were also implemented in *CNS* (Brünger *et al.*, 1998 ▸), including correlation coefficients on structure-factor intensities (target="f2f2"), structure-factor amplitudes (target="f1f1"), normalized structure-factor amplitudes (target="e1e1") and the crystallographic *R* value (target="resid"), with the default being the original correlation coefficient on normalized structure-factor intensities (target="e2e2").

To provide a similar functionality to PC refinement, our software *Phaser* (McCoy *et al.*, 2007 ▸) has been extended to allow refinement when only the orientation is known, using the maximum-likelihood framework (Read, 2001 ▸). The resulting maximum-likelihood *gyre* refinement strategy optimizes the signal from fragments with low σ_A_ (Read, 1986 ▸) and includes correction factors for measurement error (Read & McCoy, 2016 ▸). The likelihood framework also allows the incorporation of information from fixed components of the structure to improve the signal in the refinement.

**Figure 1:**
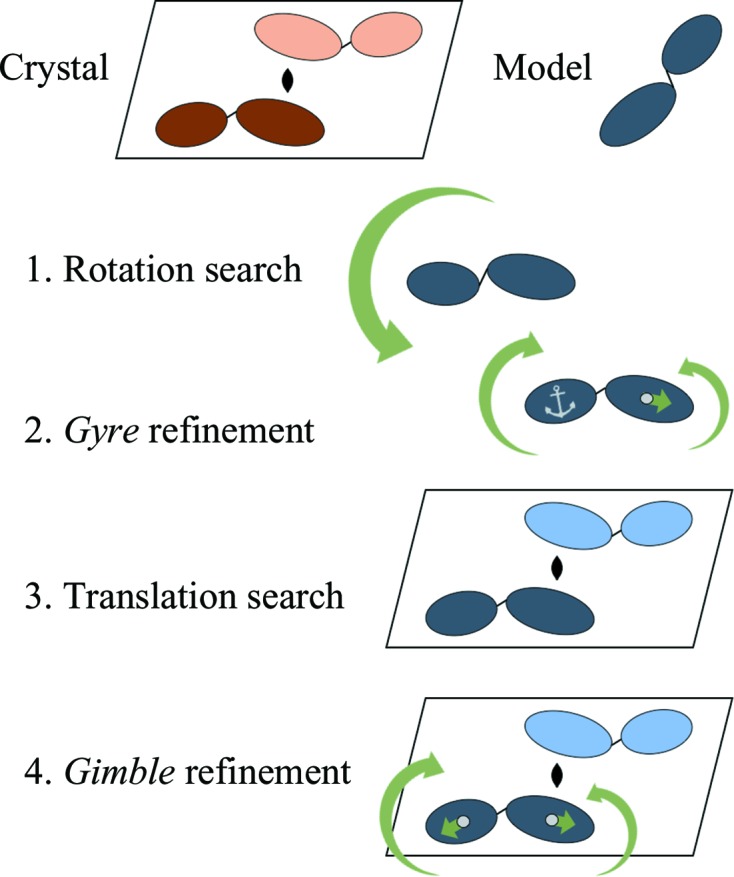
Schematic of the generalized *gyre* and *gimble* molecular-replacement protocol. Adapted from Fig. 2 in Brünger (1993 ▸). The anchor symbol indicates that the centre or mass of the domain is fixed during refinement.

To link *gyre* refinement with standard refinement against the maximum-likelihood translation/refinement-function target, *gimble* refinement (*c.f. Jabberwocky*; Carroll, 1871 ▸) has also been implemented, which similarly divides the model coordinates into rigid-body fragments, but for refinement against the translation-function/refinement maximum-likelihood function. *Gimble* refinement is not based on novel principles; it is simply a re-implementation of *Phaser*’s rigid-body refinement developed for ease of scripting. Fig. 1 ▸ shows a schematic of the *gyre* and *gimble* procedure.

To test *gyre* and *gimble*, we chose the test case for PC refinement distributed with *CNS*: solution of the Fab(26-10)–digoxin complex using Fab HyHel-5 as the model (Brünger, 1991 ▸). At the time of publication, this was a very challenging molecular-replacement problem. The challenges arise owing to the differences in the Fab hinge angle, defined as the angle between the pseudo-twofold axes of symmetry of the V_L_–V_H_ (V) and C_L_–C_H1_ (C) domain pairs, which is 161.1° for HyHel-5 and 171.5° for Fab(26-10). There are two copies of Fab(26-10) in the asymmetric unit, termed molecule *A* (chains *A* and *B*) and molecule *B* (chains *C* and *D*) by the order of identification by molecular replacement with PC refinement (Brünger, 1991 ▸). Mirroring the original study with PC refinement, the convergence of *gyre* and *gimble* for Fab(26-10) was investigated for introduced hinge-angle perturbations in HyHel-5.

There are other well established and viable approaches to molecular replacement with *Phaser* when there is a hinge motion between the model and target, such as that seen in Fab elbow angles (McCoy, 2017 ▸). *Gyre* refinement has not been developed to displace these methods, but rather for use in the context of fragment-based molecular replacement, where libraries of small fragments of structure (however derived) sample conformational space widely and where many molecular-replacement trials are performed in parallel. We specifically discuss the applications of *gyre* refinement in *ARCIMBOLDO_SHREDDER* (Millán *et al.*, 2018).

## 2. Methods   

### 2.1. Maximum-likelihood *gyre* function   

The rotation likelihood target (Read, 2001 ▸) has recently been recast to include a bias-free correction for experimental error (LLGI; Read & McCoy, 2016 ▸), and this is the basis of the *gyre* refinement target. At each orientation during *gyre* refinement, the amplitudes (but not the phases) of the structure factors of the symmetry-related copies (*s*) of the molecular-replacement fragments (*r*) oriented (but not positioned) in the unit cell can be calculated, giving a set of normalized structure-factor amplitudes {*E*_*r*,*s*_} for the rotating components. In addition, other components of the asymmetric unit may be fixed, giving a phased normalized structure-factor amplitude, which may represent the sum of a number of molecular transforms with known relative phase (*E_f_*). The probability distribution is given by a random walk in recip­rocal space. For the derivation of the maximum-likelihood rotation function, the random walk is considered to start from one of the contributions to the total structure factor, with the relative phases of the other contributions being unknown (Storoni *et al.*, 2004 ▸; Read, 2001 ▸), giving a Rice distribution. The variance of the structure-factor distribution of the remaining *E* values is smallest if the fixed structure factor is that with the largest amplitude of the set. However, structure-factor lengths change during the course of *gyre* refinement, and so the identity of the largest structure factor also changes, which would lead to instability in the minimization if not accounted for throughout refinement. To simplify the algorithm, no structure-factor contribution is fixed in *gyre*: the refinement target is a Wilson distribution. This is theoretically justified because the Wilson distribution rapidly becomes a good approximation to the Rice distribution as the number of structure factors increases, and thus is a good theoretical approximation for *gyre* refinement for all cases except those with both *P*1 crystal symmetry and only a few independently rotating fragments. Note that the Wilson distribution has been used as an approximation to the Rice distribution in *Phaser* since the inception of *Phaser*, as it is the basis for the derivation of the likelihood-enhanced fast rotation function (Storoni *et al.*, 2004 ▸). In practice, the Wilson approximation to the rotation function gives good results even in *P*1 and with a single rotating fragment.

The rotations and relative translations of the model fragments are optimized with respect to the LLGI target [equations (19*a*) and (19*b*) in Read & McCoy (2016) ▸],
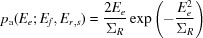
for acentric reflections and
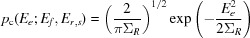
for centric reflections, where
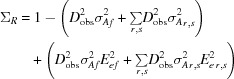
and *f* refers to the fixed models, *r* to the rotating models and *s* to the symmetry-related molecules in the unit cell, and *E_e_* and *D*_obs_ are defined as in Read & McCoy (2016 ▸): *E_e_* is the effective *E*, representing information derived from the observed normalized intensity, and *D*_obs_ represents the reduction in correlation between observation and *E_e_* arising from experimental error. Analytic derivatives are calculated with respect to rotation, translation and σ_A_ of the components.

### 2.2. Parameterization   

Rotational refinement of the coordinates of each fragment is parameterized as three angular perturbations around orthogonal directions in space and about the centre of mass of the model. Likewise, the positional refinement is parameterized as perturbations of the centre of mass in orthogonal directions in space. Since only the relative position of the fragments can be refined against the rotation likelihood target, the centre of mass of the heaviest fragment is arbitrarily fixed. Parameterization in terms of orthogonal perturbations gives good convergence in the minimizer for the small perturbations expected from the nature of the problem and enforced by the restraints. This parameterization also allows straightforward reporting of the changes in orientation and position of the fragments during the refinement. As implemented in *Phaser*, individual atomic coordinates cannot be refined against the *gyre* target function, since there are no geometry restraints. The σ_A_ (a function of the VRMS) of the fragments is also refined, in a procedure analogous to that described previously (Oeffner *et al.*, 2013 ▸).

### 2.3. Restraints   

The rotations and translations may be restrained to the unperturbed orientation and position by a harmonic restraint. By default, the rotation is restrained with a weak standard deviation of 25°, which prevents very small fragments with little contribution to the scattering from spinning away from their initial orientations. By default, the translation is restrained with a tight standard deviation of 2 Å, which only allows the position to change when the signal for the translation is strong. The appropriate restraints to use in any given case will be dependent on the size of the fragments and the resolution of the data. Restraint terms may be set globally, per refinement cycle or per fragment (McCoy *et al.*, 2009 ▸).

### 2.4. Error estimation   

The σ_A_ estimation has a resolution-independent term, which is determined by the fraction of the total scattering represented by the model (*f*_m_), and a resolution-dependent term, which decreases with increasing r.m.s.d., so that poorer models down-weight the high-resolution data. Estimates of the r.m.s.d. and *f*_m_ are therefore required to estimate σ_A_. The optimal estimate of the r.m.s.d. for proteins has been developed and is a function of the sequence identity between model and target and the number of residues in the target (Oeffner *et al.*, 2013 ▸). Appropriate estimates of r.m.s.d. have been shown to be decisive in solving difficult molecular-replacement cases (Oeffner *et al.*, 2013 ▸). When the whole model can be superimposed on the target, the estimate of *f*_m_ is only dependent on the estimate of the asymmetric unit contents.

The conversion of the r.m.s.d. and *f*_m_ to an appropriate σ_A_ as described above assumes no systematic shift of a subset of atoms between the model and target; however, *gyre* refinement has been developed for use in precisely such cases. When there is a systematic shift of a subset of atoms between the model and target, the model structure factor can be thought of as the sum of two structure factors, one of which (that corresponding to the subset of atoms correctly oriented) contributes much more to the molecular-replacement signal than the other. However, only a total structure factor is calculated and an overall σ_A_ applied. The appropriate σ_A_ for the total structure factor will be lower than that expected were the whole model to be contributing strongly to the signal, but by an unknown amount.

Not only will the appropriate initial estimation of σ_A_ for the model be extremely problematic in *gyre* refinement, the σ_A_ should increase rapidly during refinement as the systematic shift in coordinates is corrected.

The problem of error estimation for *gyre* refinement is confronted in several ways. The default *Phaser*-implemented function (Oeffner *et al.*, 2013 ▸) to estimate r.m.s.d. from sequence identity and model molecular weight should not be used. Rather, an explicit r.m.s.d. should be set for the model. Further, since the appropriate value is unknown, it is often necessary to trial different r.m.s.d. values. To accommodate some of the changes in the errors during refinement, the r.m.s.d.-associated variance (VRMS) is refined in *gyre* refinement. The value of *f*_m_ can be lowered by the use of the ‘search occupancy’ parameter (McCoy *et al.*, 2009 ▸), which reduces the scattering from the model by a scale factor. Although theor­etically possible, refinement of *f*_m_ is not implemented in *Phaser*. Finally, since the signal from the rotation function is likely to be reduced by the error in the estimation of σ_A_, more rotation-function peaks should be passed to the *gyre* refinement than would be passed to a standard translation function by default.

### 2.5. Implementation   

From *Phaser*-2.7.12, *gyre* and *gimble* refinement can be invoked from the scripting interface or the Python interface (McCoy *et al.*, 2009 ▸). The results described here refer to *Phaser*-2.8.1 and above. *Gyre* is performed with the GYRE mode and *gimble* with the GIMBLE mode (McCoy *et al.*, 2009 ▸).

**Figure 2:**
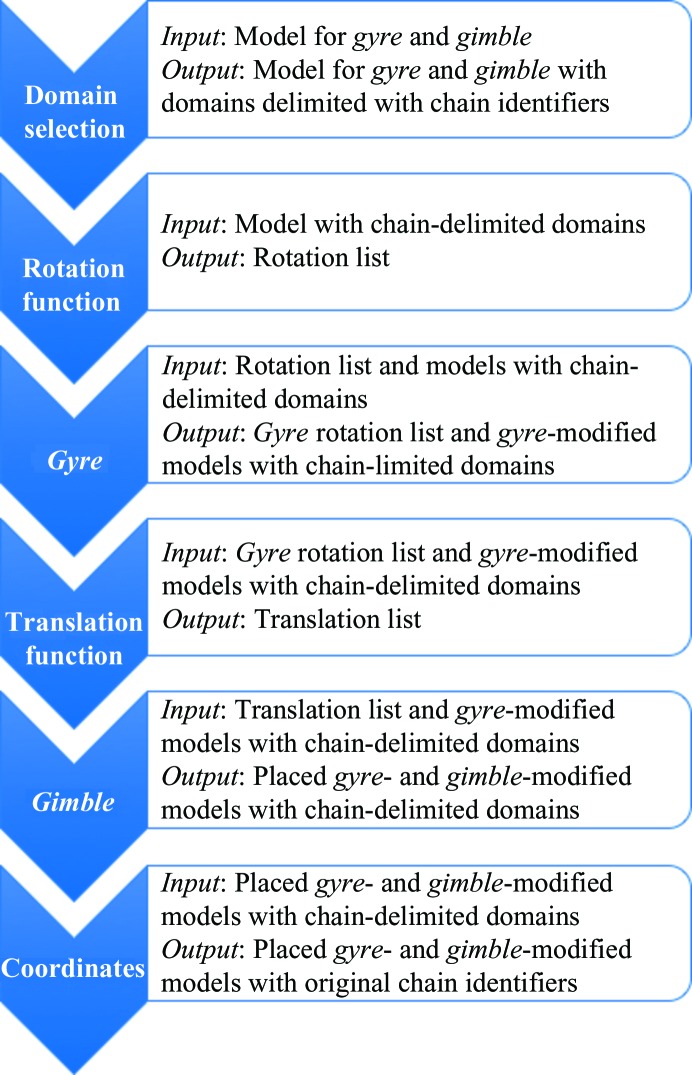
Flow diagram for *gyre* and *gimble* refinement as implemented in *phenix.gyre_and_gimble*. *Gyre* refinement takes the rotation list from a standard *Phaser* rotation function and, for each orientation, refines the orientation and relative positions of the domains. One coordinate file is output for each input orientation. The corresponding rotation list output from *gyre* refinement has all of the orientation angles set to zero, with the coordinates to which each orientation refers being different. This is in contrast to a standard rotation list, where the coordinates for each trial rotation are the same and it is the orientations that differ. After the translation function, *gimble* refinement modifies the positions and orientations of the fragments by refinement against the LLGI target, and the final oriented, placed and perturbed coordinates are written out. The *phenix.gyre_and_gimble* implementation optimizes the placement of domains for a single copy of a model in the asymmetric unit. Other models can be placed in the asymmetric unit using standard molecular replacement or, if conformational change is suspected in further components, further *gyre* and *gimble* procedures.

Fig. 2 ▸ shows the flow diagram for the *PHENIX* (Adams *et al.*, 2011 ▸) tool *phaser.gyre_and_gimble*. Rigid-body domains for the *gyre* and *gimble* refinements are defined using the *X-PLOR*/*CNS*/*PHENIX*/*PyMOL* atom-selection syntax (Brünger, 1992 ▸). The script checks that the fragment selections are mutually exclusive and warns the user of atoms that are not assigned to fragments. The domain selection can be checked independently with *phaser.gyre_pdb_tool*, which outputs the coordinate file with chain identifiers altered as requested by the user. During *phaser.gyre_and_gimble*, one copy of the molecular-replacement search model undergoes *gyre* and *gimble* refinement and is placed in the asymmetric unit. See Appendix *A* [App appa].

Phaser’s *gyre* and *gimble* functionality is also available separately as *Phaser* modes (MODE GYRE/MODE GIMBLE; McCoy *et al.*, 2009 ▸) and can be used to build scripts for specific cases either through the scripting or the Python interface. Domains for *gyre* and *gimble* in the separate *Phaser* modes are demarcated by the assignment of different chain identifiers. The chain identifiers can be edited in the coordinate file *via* a text editor, using graphical selection tools such as *Coot* (Emsley *et al.*, 2010 ▸) or automatic domain-demarcating procedures such as *Phaser* SCEDS (McCoy *et al.*, 2013 ▸).

## 3. Results   

We chose the solution of the Fab(26-10)–digoxin complex using Fab HyHel-5 as a model to test *gyre* and *gimble* (Brünger, 1991 ▸). The Fab(26-10) structure is deposited in the Protein Data Bank as PDB entry 1igj, with experimental data representing the twinned data described in Brünger (1991 ▸), whereas the data distributed with *CNS* are detwinned (Brünger, 1991 ▸; Jeffrey *et al.*, 1993 ▸). We chose to use the detwinned data, as these were used in the original study, but rather than truncating the data at different resolutions, we used variation of the estimated r.m.s.d. between the model and target to give different resolution-dependent weighting of the structure factors in the likelihood function.

**Figure 3:**
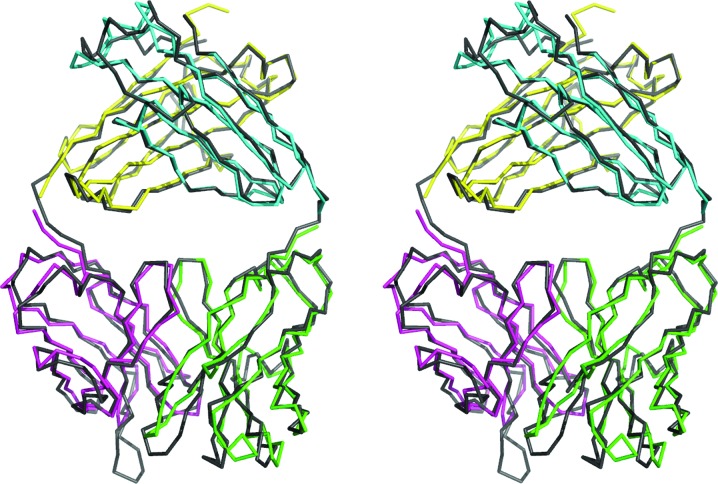
Stereoview of V_H_, V_L_, C_L_ and C_H1_ domains of PDB entry 2hfl superimposed on the corresponding domains of PDB entry 1igj. The r.m.s.d. between optimally aligned 2hfl and 1igj is 1.1 Å over 214 core residues for the variable domains (V_L_ and V_H_) and 0.95 Å over 198 core residues for the constant domains (C_L_ and C_H1_), as calculated by *SSM* (Krissinel & Henrick, 2004 ▸) in *Coot* (Emsley *et al.*, 2010 ▸). Breaking the model and target into the four antibody domains further lowered the r.m.s.d. slightly: V_L_, 0.98 Å (102 residues); V_H_, 1.1 Å (109 residues); C_L_, 0.80 Å (103 residues); C_H1_, 0.95 Å (93 residues). These are the minimum r.m.s.d. values obtainable by molecular replacement.

The *CNS*-distributed HyHel-5 coordinates are taken from the structure of HyHel-5 in complex with lysozyme, which was deposited in the Protein Data Bank as PDB entry 2hfl (Sheriff *et al.*, 1987 ▸) and was subsequently superseded by PDB entry 3hfl (Cohen *et al.*, 1996 ▸) and by PDB entry 1yqv (Cohen *et al.*, 2005 ▸). We chose to use the coordinates of the now obsolete PDB entry 2hfl for this study. Unlike the original structure solution, where the *B* factors of the search model were doubled, no modification of the deposited *B* factors was performed and nor were any of the currently recommended structure-preparation methods used (Bunkóczi & Read, 2011 ▸; Schwarzenbacher *et al.*, 2004 ▸). The ideal superposition of 2hfl on 1igj is shown in Fig. 3 ▸.

### 3.1. Standard molecular replacement   

**Figure 4:**
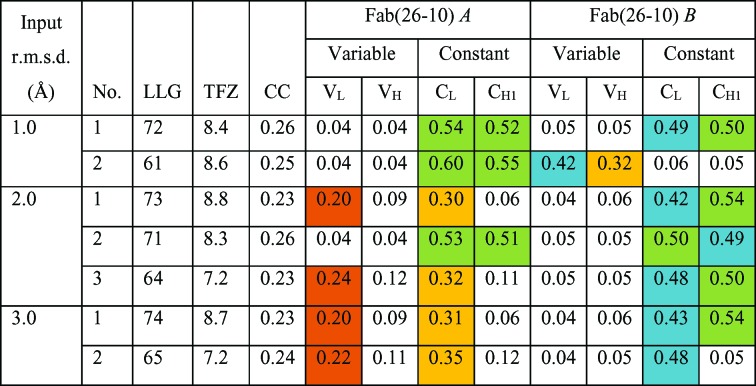
Molecular-replacement solutions generated by *Phaser* for PDB entry 1igj solved using 2hfl without *gyre* and *gimble* refinement. The overall CC (map CC for *Phaser*-generated map coefficients FWT and PHWT for MR placement and *Phaser*-generated map CC for target 1igj) is low for all solutions: between 0.23 and 0.26. Different combinations of domains (domain H, H 1–113; domain K, H 113–223; domain L, L 1–106; domain M, L 107–200) overlie the structure well for each solution. CC per Fab domain is shown coloured by value: CC > 0.50, green; CC > 0.40, blue; CC > 0.30, yellow; CC > 0.20, orange.

We confirmed that structure solution by molecular replace­ment is still not straightforward, despite the improvements in crystallographic methods since 1991. *Phaser* does not produce a solution clearly separated from noise for any of three initial estimates of the model-to-target r.m.s.d. (1, 2 and 3 Å). After accounting for origin shifts and crystallographic symmetry, it could be seen that the top solutions represent different partial overlaps of 2hfl with 1igj, or indeed no significant overlap (Fig. 4 ▸). More sophisticated protocols for molecular replacement with *Phaser* (McCoy, 2017 ▸) are able to give clear and accurate domain placements.

### 3.2. *Gyre* and *gimble*   

**Figure 5:**
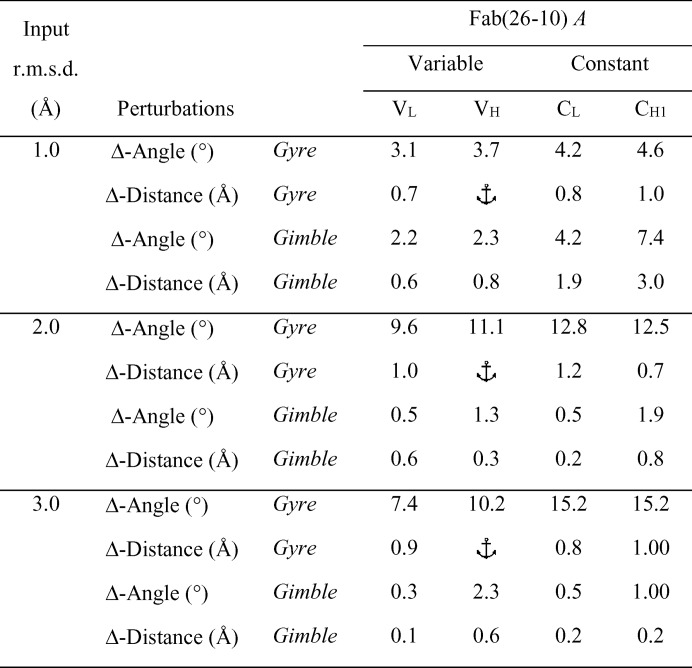
*Gyre* and *gimble* rotations and translations for PDB entry 1igj solved using 2hfl. The solution corresponds to molecule *A* in PDB entry 1igj.

**Figure 6:**
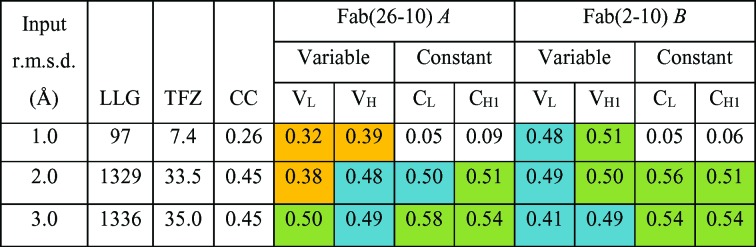
Molecular-replacement solutions generated by *Phaser* for PDB entry 1igj solved using 2hfl following *gyre* and *gimble*. CC per domain (domain H, H 1–113; domain K, H 113–223; domain L, L 1–106); domain M, L 107–200) is shown coloured by value: > 0.50, green; > 0.40, blue; > 0.30, yellow. With an input r.m.s.d. of 1.0 Å the molecular-replacement solution is no better than the standard molecular-replacement solution (Fig. 4 ▸), but for an r.m.s.d. of 2.0 or 3.0 Å the overall CC is 0.45, that of the unperturbed aligned structure (Figs. 2 ▸ and 4 ▸).

Since the original study (Brünger, 1991 ▸) allowed V_L_, V_H_, C_L_ and C_H1_ to move independently, the V_L_, V_H_, C_L_ and C_H1_ domains of 2hfl were demarcated as different domains (Appendix *A* [App appa]). As for standard molecular replacement, three initial estimates of the r.m.s.d. were used: 1, 2 and 3 Å. The 2hfl structure was subjected to *gyre* and *gimble* (Fig. 5 ▸), and the output coordinates were used for standard molecular replace­ment, searching for two copies of the perturbed Fab (Fig. 6 ▸). The input r.m.s.d. is shown to be an important parameter for success with *gyre* and *gimble*. Only input r.m.s.d. values of 2 and 3 Å gave very high LLG and TFZ values and resulted in all antibody domains having high density correlation to the 1igj density.

**Figure 7:**
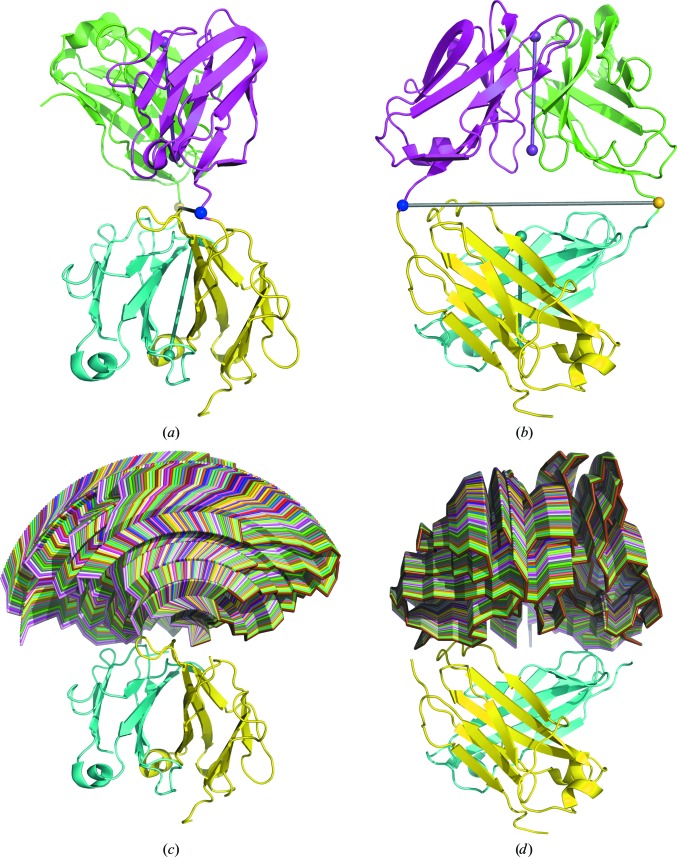
PDB entry 2hfl perturbed ±35° in 1° increments from optimal superposition on PDB entry 1igj. (*a*) The purple and cyan dumbbells pass through the centres of mass of the variable and constant domains, respectively, of each Fab, showing the pseudo-twofold axis. The grey dumbbell shows the axis of rotation, with the residues used to split the domains shown in blue for the light chain and yellow for the heavy chain. (*b*) shows (*a*) rotated through 90°. (*c*) The perturbed structures of Fv shown in ribbon representation, with each perturbation in a different colour, from the same view as (*a*). (*d*) shows (*c*) rotated through 90° from the same view as (*b*). The figure and perturbed coordinates were generated with *PyMOL* (DeLano, 2002 ▸)

To test the convergence of the *gyre* refinement, we followed the original study and looked at the behaviour as a function of the elbow-angle difference between modified Fab HyHel-5 structures and the correct Fab(26-10) structure. Firstly, an artificial structure of HyHel-5 was generated with the C and V domains superimposed on the C and V domains of Fab(26-10), representing the ideal model (Fig. 7 ▸). The elbow angle of HyHel-5 was then modified by rotating the V domain around the hinge axis, passing though residue 106 of the light chain and residue 116 of the heavy chain, using a Python script based on the elbow.py script available from the *PyMOL* wiki (DeLano, 2002 ▸). Again, three initial estimates of the r.m.s.d. were used, 1, 2  and 3 Å, and again this is shown to be an important parameter in the convergence (Fig. 5 ▸).

**Figure 8:**
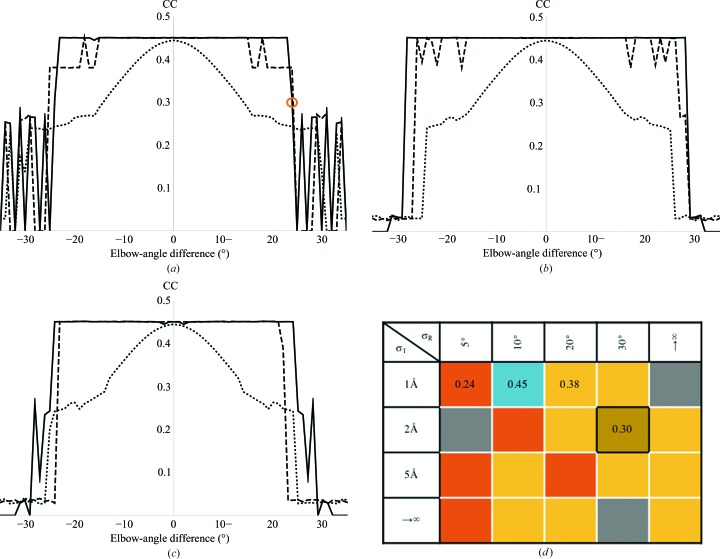
Map correlation coefficient (CC; map CC for *Phaser*-generated map coefficients FWT and PHWT for MR placement and *Phaser*-generated map CC for target 1igj) for PDB entry 1igj solved using 2hfl pre-aligned with 1igj as shown in Fig. 3 ▸ and perturbed as shown in Fig. 7 ▸. Solid lines show the CC after *gyre* and *gimble*, dotted lines show the CC for standard molecular replacement and dashed lines show the CC for *gimble* refinement only. The rotational restraint was 30° and the translational restraint was 2 Å. The input r.m.s.d. values were (*a*) 1.0 Å, (*b*) 2.0 Å and (*c*) 3.0 Å. (*d*) Convergence of the *gyre*-and-*gimble*-refined solution as a function of standard deviation of the rotational (σ_R_) restraints and translational (σ_T_) restraints (where →∞ indicates unrestrained) for 2hfl with a perturbation angle of +24°. Correlation coefficients are shown coloured by value (CC = 0.45, blue; CC = 0.38, yellow; CC = 0.30, gold; CC = 0.24, orange); grey indicates that molecular replacement failed. With *gimble* refinement only CC = 0.38 and with standard molecular replacement CC = 0.24. The black box in (*d*) indicates the value circled in orange in (*a*).

The convergence of *gyre* and *gimble* with respect to the elbow-angle difference of the Fab was much greater than for PC refinement (Figs. 8 ▸ *a*, 8 ▸ *b* and 8 ▸ *c*). Whereas the results presented in Fig. 8 of Brünger (1991 ▸) indicated that the solution would converge from an elbow-angle difference of 10°, with the optimal parameters for *gyre* and *gimble* the solution converged from +28/−29° (Fig. 8 ▸ *b*).

To determine the contribution to the increased radius of convergence from the *gyre* refinement, molecular replacement was performed including *gimble* refinement but omitting the *gyre* step (Figs. 8 ▸ *a*, 8 ▸ *b* and 8 ▸ *c*). The radius of convergence was higher than with the PC target, as expected from the higher sensitivity of maximum-likelihood target functions to the correct placement over Patterson target functions (Read, 2001 ▸). However, the *gyre* refinement was shown to add significantly to the radius of convergence, particularly at the lower input r.m.s.d. values.

The convergence of *gyre* refinement is heavily dependent on the strength of the harmonic restraints. The results of different restraint values on the convergence from a hinge angle of 24° are shown in Fig. 8 ▸(*d*). For the test case, *gyre* convergence was better when the translation was restrained. However, appropriate restraint values are case-dependent (results not shown), most likely determined by the size of the fragments and the resolution of the data. The strong dependence of convergence on restraint values indicates that a range of restraint values should be used to achieve optimal results.

## 4. *ARCIMBOLDO_SHREDDER*   

*Gyre* refinement has been incorporated into *ARCIMBOLDO_SHREDDER* (Sammito *et al.*, 2014 ▸; Millán *et al.*, 2018 ▸). *ARCIMBOLDO_SHREDDER* performs highly parallel and systematic molecular-replacement searches using a library of small structure motifs derived from a homologous structure (Sammito *et al.*, 2013 ▸) and analyses the results to extract information from the persistence of solutions for different fragments among the noisy rotation-function results from *Phaser*. Potential molecular-replacement solutions are passed to *SHELXE* (Sheldrick, 2010 ▸) for density modification and model building, with the prospect that any correctly placed fragments can be expanded into a full structure.

The small fragments of structure that are generated by *ARCIMBOLDO_SHREDDER* commonly contain secondary-structure elements that differ slightly in orientation and position between the model and target, and hence the disposition of the secondary-structure elements can be improved by *gyre* refinement. Apart from improving the model, *gyre* refinement can also give an early indication of which rotations are more likely to align with correct placements, and hence which rotations should be prioritized for passing to the subsequent stages of phasing. The convergence tests described here indicate that there is better convergence when the translational component of *gyre* is restrained to the input position. This agrees with the results from *ARCIMBOLDO_SHREDDER*, where small fragments may wander far from the starting position if not restrained.

*ARCIMBOLDO_SHREDDER* approaches the problem of error estimation by performing a series of *gyre* refinements gradually reducing the expected r.m.s.d. of the fragments and performing VRMS minimization, which is highly effective in increasing the radius of convergence.

The introduction of *gyre* refinement in *ARCIMBOLDO_SHREDDER* has been instrumental in a number of structure solutions to date, and will be described elsewhere (manuscript in preparation).

## 5. Discussion   

Hinge motions between domains may still confound molecular replacement, because it is not possible to simultaneously overlay all domains in the model on the target. The molecular-replacement signal is degraded both by the smaller fraction scattering of the total that can be superposed on the target and by the noise introduced by the necessity of incorrectly placing a substantial fraction of the atoms. When there is a hinge motion between the model and target, *Phaser* frequently finds several different mutually exclusive solutions, where different combinations of domains are correctly overlaid on the target or, for small hinge motions, a solution that represents a compromise fit of all domains to the target. These solutions, although in some way correct, can be challenging to carry forward to model building and refinement; *phenix.morph_model* (Terwilliger *et al.*, 2013 ▸) and *REFMAC*’s jelly-body refinement (Murshudov *et al.*, 2011 ▸) can be very helpful in this regard.

Rotational refinement has been available in *Phaser* from its inception by using the brute-force ‘rotate around’ protocol (McCoy *et al.*, 2009 ▸). Rotations on a grid and within a restricted range of angles about a central orientation are scored against the maximum-likelihood (Rice) rotation function. The ‘rotate around’ protocol has been most usefully applied when the orientation and position of a small and/or weakly scattering domain can be inferred from the placement of a larger and/or more strongly scattering domain. The ‘rotate around’ protocol can be used to optimize the orientation of this domain, in conjunction with the analogous ‘translate around’ protocol for optimizing the position. Unlike *gyre* refinement, this protocol can only be used to optimize the orientation and position of one fragment at a time, and does not include σ_A_ refinement.

In cases of a hinge motion being present and where the data are numerous, splitting the model may yield domains that retain a significant LLGI and hence signal in molecular replacement. These domains can be searched for by sequential addition, exploiting the strength of the maximum-likelihood target in using information from already oriented and positioned components in the asymmetric unit to increase the signal in the search for the second and subsequent components. However, if the data do not extend to very high resolution, decreasing the fraction scattering of the model is likely to reduce the LLGI below the level of significance. These are the cases for which *gyre* refinement is most likely to assist structure solution as an extension of standard molecular replacement.

Molecular replacement using fragments of distant homologues is now established as a viable method for solving protein structures. The method relies on the fragments having low r.m.s.d. in atomic coordinates between the model and target to offset the low fraction of the total scattering that they represent. When correctly placed, small motifs of secondary structure, such as a helix–turn–helix or a three-stranded β-sheet, can act as seeds for structure expansion with density-modification methods. However, whether they are derived from structures with sequence identity to the target or from a general structure-motif library, the relative angles and positions of these secondary-structure elements are likely to differ by a few degrees and ångströms between the model and target. Since these approaches use large libraries of fragments and parallel molecular-replacement trials, any early indications that phasing is succeeding can be used to reduce the number of trials necessary for structure solution. By increasing the signal from molecular replacement at the rotation-function step, *gyre* refinement has been shown to both reduce the computation time and increase the success rates (Millán *et al.*, 2018 ▸).

The use of *gimble* refinement is not restricted to use in tandem with *gyre* refinement. The radius of convergence of the *Phaser* rigid-body refinement algorithm, for refining placed components at the end of molecular replacement, is very robust. Crystallographers may find that *gimble* refinement of appropriately annotated chains within a solution will accelerate model building and refinement because the process is started from a better model and a better phased electron-density map.

Like Brünger’s PC refinement, resolution is shown to be important in the convergence of *gyre* refinement. High estimated r.m.s.d. values, which down-weight the high-resolution terms, increased the radius of convergence. We would advise performing *gyre* refinement with a range of r.m.s.d. values well above those estimated from the sequence identity (Oeffner *et al.*, 2013 ▸). However, the effectiveness of this strategy will depend on the resolution limit of the data and may not be ideal when the resolution is low. Altering the σ_A_ estimations at the four steps of the *gyre* and *gimble* procedure may better estimate the errors at the different stages. Specialized strategies, such as those employed by *ARCIMBOLDO_SHREDDER*, will be even more effective. Just as the optimal r.m.s.d. is unknown in advance, so the appropriate standard deviations for the rotation and translation perturbation restraints are also unknown in advance, and we would advise performing *gyre* refinement with a range of restraint values, not just those imposed by default.

The proven advantages of the maximum-likelihood framework over Patterson methods for molecular replacement, and the results presented here, lead us to expect that *phaser.gyre_and_gimble* (see Appendix *B* [App appb]) will prove to be at least as useful as PC refinement to crystallographers attempting the solution of challenging molecular-replacement cases.

## Appendix A *phaser.gyre_pdb_tools*

*phaser.gyre_pdb_tools* is available in the *PHENIX* software package (Adams *et al.*, 2010 ▸).

### A1. PHIL parameters

The following script may be used to generate the domain definitions used in this study (Echols *et al.*, 2012 ▸).

### A2. Command-line interface

The following script may be used to generate the domain definitions used in this study. Note that the command-line interface does not give the user control of the chain identifiers of the atom selection on output.

## Appendix B *phaser.gyre_and_gimble*

*phaser.gyre_and_gimble* is available in the *PHENIX* software package (Adams *et al.*, 2002 ▸). The template PHIL file is shown below (Echols *et al.*, 2012 ▸).
